# Characteristics of Beverage Consumption Habits among a Large Sample of French Adults: Associations with Total Water and Energy Intakes

**DOI:** 10.3390/nu8100627

**Published:** 2016-10-11

**Authors:** Fabien Szabo de Edelenyi, Nathalie Druesne-Pecollo, Nathalie Arnault, Rebeca González, Camille Buscail, Pilar Galan

**Affiliations:** 1 Sorbonne Paris Cité Epidemiology and Statistics Research Center (CRESS), Inserm U1153, Inra U1125, Cnam, Paris 13 University, Nutritional Epidemiology Research Team (EREN), Bobigny F93017, France; f.szabo@eren.smbh.univ-paris13.fr (F.S.d.E.); n.pecollo@eren.smbh.univ-paris13.fr (N.D.-P.); n.arnault@eren.smbh.univ-paris13.fr (N.A.); r.gonzalez@eren.smbh.univ-paris13.fr (R.G.); c.buscail@eren.smbh.univ-paris13.fr (C.B.); 2 Public Health Department, Avicenne Hospital, Bobigny F93017, France

**Keywords:** nutrients, total water intake, energy intake, beverages, France

## Abstract

Background: Adequate hydration is a key factor for correct functioning of both cognitive and physical processes. In France, public health recommendations about adequate total water intake (TWI) only state that fluid intake should be sufficient, with particular attention paid to hydration for seniors, especially during heatwave periods. The objective of this study was to calculate the total amount of water coming from food and beverages and to analyse characteristics of consumption in participants from a large French national cohort. Methods: TWI, as well as contribution of food and beverages to TWI was assessed among 94,939 adult participants in the Nutrinet-Santé cohort (78% women, mean age 42.9 (SE 0.04)) using three 24-h dietary records at baseline. Statistical differences in water intakes across age groups, seasons and day of the week were assessed. Results: The mean TWI was 2.3 L (Standard Error SE 4.7) for men and 2.1 L (SE 2.4) for women. A majority of the sample did comply with the European Food Safety Authority (EFSA) adequate intake recommendation, especially women. Mean total energy intake (EI) was 1884 kcal/day (SE 1.5) (2250 kcal/day (SE 3.6) for men and 1783 kcal/day (SE 1.5) for women). The contribution to the total EI from beverages was 8.3%. Water was the most consumed beverage, followed by hot beverages. The variety score, defined as the number of different categories of beverages consumed during the three 24-h records out of a maximum of 8, was positively correlated with TWI (*r* = 0.4); and with EI (*r* = 0.2), suggesting that beverage variety is an indicator of higher consumption of food and drinks. We found differences in beverage consumptions and water intakes according to age and seasonality. Conclusions: The present study gives an overview of the water intake characteristics in a large population of French adults. TWI was found to be globally in line with public health recommendations.

## 1. Introduction

Hydration status results from a tightly regulated balance between water intake and loss. It is important to equilibrate input and output because even mild dehydration can lead to a reduction of physical and cognitive performances [1,2]. A more severe dehydration status can have a very significant impact on health, especially in elderly people [3]. On the long term, some effects of hydration status on chronic diseases have been shown or suggested, with different levels of evidence [4,5].

Water requirements vary depending on environmental factors such as heat, salt intake, lifestyle (physical activity) and inter-individual variability. Water intake is fulfilled by both water contained in solid foods (20%–30%) and water from beverages and drinking water (70%–80%) [5,6,7].

While depending on eating and drinking habits, adaptation of the water intake to match the variation in water loss is mostly driven by thirst [8,9].

Some countries and public organizations have proposed water intake recommendations for the general public. Due to the large inter-individual variability, those recommendations struggle to give adequate reference values for total water intake. European Food Safety Authority (EFSA) proposed Dietary Reference Values (DRV) for Adequate Intake of Water (AI) for men and women of 2.5 L and 2.0 L [6].

Few data are available concerning the total amount of water coming from food or beverages and characteristics of beverage consumption in the French general population.

## 2. Materials and Methods

### 2.1. Data Collection

Data used in this study were collected using the Nutrinet-Santé cohort. The Nutrinet-Santé Study is a large web-based prospective observational cohort including adult volunteers aged 18 years or older, launched in France in May 2009 with a scheduled follow-up of 10 years. The Nutrinet-Santé study has been described in detail elsewhere [10]. The Nutrinet-Santé study was conducted according to guidelines laid down in the Declaration of Helsinki and was approved by the International Research Board of the French Institute for Health and Medical Research (IRB Inserm Paris, France No. 0000388FWA00005831) and the “Comité National Informatique et Liberté” (CNIL Paris, France No. 908450 and No. 909216). Electronic informed consent was obtained from all subjects.

At baseline, socio-demographic data including age, gender, education, income, occupational category, and household location, as well as lifestyle (smoking status, physical activity), height, weight and practice of restrictive diet were self-reported. Leisure time physical activity was assessed using the French short form of the International Physical Activity Questionnaire (IPAQ), self-administered online [11,12,13]. Body mass index (BMI) was assessed using self-reported height and weight.

Dietary data were also collected at baseline using three 24-h records, randomly distributed within a two-week period, including two week days and one weekend day. Participants reported all foods and beverages consumed throughout the day: breakfast, lunch, dinner and all other occasions.

In the present study, daily mean food and beverage consumptions were calculated for each participant having completed the three 24-h records, with a weighting on the type of day (week or weekend day). Identification of underreporting participants was based on the validated and published method proposed by Black [14] using Schofield equations for estimating resting metabolic rate [15]. In addition, we eliminated subjects with anomalous values for EI (men <800 or >4000 and women <500 or >3500 kcal/day) [16]. Serving sizes were estimated using purchase unit, household unit and photographs, derived from a previously validated picture booklet [17].

Participants were asked at the end of each 24-h records whether they did not forget any food intake, including snacking and beverages, and had then the possibility to add it to the records.

A total of 94,939 participants were included in this analysis.

### 2.2. Data Preparation and Analysis

EI and TWI were calculated through the NutriNet-Santé food composition table including more than 2000 food products [18]. Contributions of food and beverages to TWI and EI were also calculated.

Beverages have been grouped into 8 categories for further analysis: (1) hot beverages included hot tea and hot coffee; (2) milk; (3) fruit & vegetable 100% juices with no added sugar (not included nectars and mix of juice and milk); (4) caloric soft drinks (included sodas, ice tea, non-alcoholic beer or liquor, sports drinks, energy drinks, nectars and mix of juice and milk, etc.); (5) diet soft drinks (without sugar as a sweetener); (6) alcoholic drinks; (7) water (include tap water and bottled water); and (8) other beverages (included soy-based beverages, high-protein beverages). Contribution of each beverage category to the total beverage consumption was computed. A variety score defined as the number of different categories of beverages consumed during the three 24-h records out of a maximum of 8 was calculated.

Five age groups were used in the study: 18–25, 26–35, 36–50, 51–64 and 65–75 years.

In order to estimate the effect of the season on beverage consumption, nutritional data were separated according to the month of the 24 h-records: December–January–February (winter), March–April–May (spring), June–July–August (summer) and September–October–November (autumn). Beverage consumptions were also calculated separately for each day of the week in order to test whether there was a trend over a week. For this calculation, no weighting on the type of day (week or weekend day) was performed.

To investigate trends over the day, consumption occasions were aggregated into 6 periods of time, approximately corresponding to breakfast (5:30 to 10:00), mid-morning (10:00 to 12:00), lunch (12:00 to 15:00); snack (15:00 to 19:00); dinner (19:00 to 22:00); and other moments.

### 2.3. Statistical Analyses

Description of the population, contribution of food and beverages to total water and energy intake and beverage consumption according to time of day and day of the week were stratified by sex. Spearman partial correlations between water intake, energy intake and beverage consumption were adjusted for age, gender, body weight and physical activity level. Crude differences in TWI and beverage consumption across age groups and across seasons were assessed by sex through ANOVA-test. Pairwise comparisons of the means across groups were assessed using *T*-tests with Bonferroni correction for multiple testing. Comparisons of the means of beverage consumption across week-end days (Saturday, Sunday) and the other days of the week were assessed by sex using *T*-tests. Crude differences in beverage consumption at 6 periods of time, across age groups were assessed through ANOVA-test. Pairwise comparisons of the means were assessed using *T*-tests with Bonferroni correction for multiple testing. Partial correlations between TWI and beverage consumption in each period of time, by gender, were adjusted for age. Beverage consumption in each period was expressed as a percentage of total consumption over 24 h. All analyses were 2-tailed with a statistical significance of *p* < 0.05. 

## 3. Results

Characteristics of the sample are presented for men and women as well as for the whole sample in Table 1. The mean age was 42.9 (0.04) (41.7 (0.05) for women and 47.3 (0.1) for men) and 78% were women; 63.7% had a post-secondary degree education level. The mean BMI was 23.8 kg/m^2^, with 9.0% of participants being obese and 21.4% being overweight.

**Figure 1:**
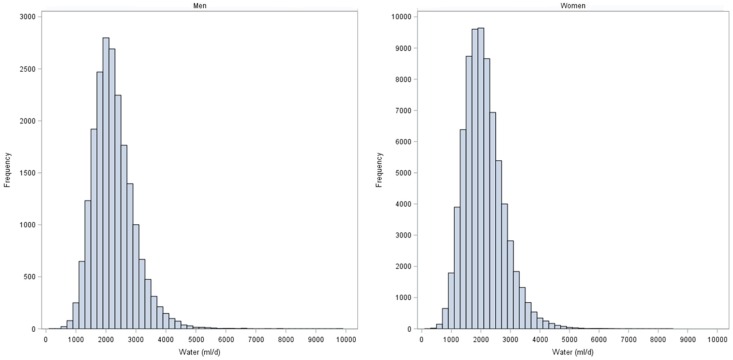
Frequency distribution of total water intake (mL/day) by gender.

The distribution of mean total water intake (TWI) (g/day), stratified by sex is shown Figure 1. The mean TWI was 2.3 L for men and 2.1 L for women, close to the EFSA “adequate intake” (AI) recommendations for adults: 2.5 L and 2 L, respectively, though lower for men and higher for women.

The contributions of food and beverages to daily EI (kcal/day) and water intake (mL/day), by gender, are presented in Table 2. Men consumed more than two times more alcoholic drinks than women (*p* < 0.0001), while women consumed more hot beverages (*p* < 0.0001). Mean total EI was 1884 kcal/day (SE 1.5), and the relative contribution to total EI from beverages was 8.3% (9.9% in men, 7.8% in women). Furthermore, 61.9% of the TWI came from beverages and 38.1% came from food. The part of the water intake coming from the beverages was lower than the EFSA estimation (70%–80% provided by the beverages and 20%–30% coming from food).

**Figure 2:**
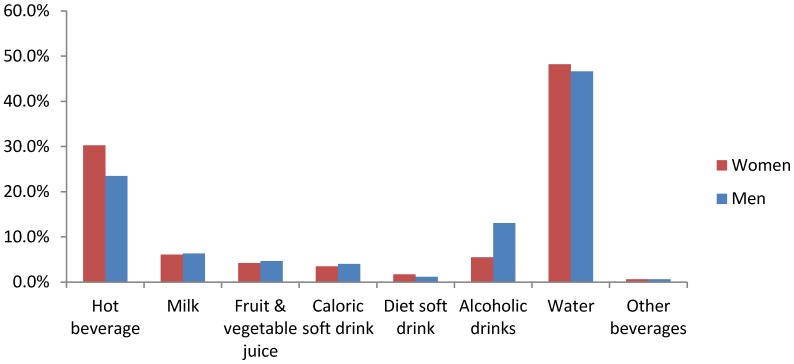
Percentage of the beverage consumption represented by each beverage category, separated by gender.

Water represented almost half of the beverage consumption over 3 days period for both men and women (48.2% for women and 46.6% for men) (Figure 2). The second most popular beverage was hot beverages (respectively 30.2% and 23.5% for women and men). Alcohol was in the third position for men (13.1%) and fourth position for women (5.5%).

TWI was highly correlated with the weight of beverages and water from beverages (*r* = 0.9) and more weakly correlated with food intake (*r* = 0.5) (Table 3). Milk and alcohol drinks had moderate correlation (*r* = 0.4 and *r* = 0.5, respectively) with total energy from beverages, while for hot beverages and diet soft drinks coefficients were the lowest values. The variety of beverages was positively correlated with beverage intake (weight, energy and water intake), suggesting that a higher diversity was associated with a higher consumption of beverages.

In Table 4, we compared the water intakes and beverage consumptions across the five age groups separately for men and women. The lowest total water intake from food and beverages was found in the 18–25 years group. Elderly people (refer here to age group 65–75 years), especially men, tend also to have lower TWI than the other age groups. For both men and women, the contributions of food and beverages to the TWI were different between elderly and other adults. Elderly people had a higher water intake from food and a lower intake from beverages than the other age groups (except with (51–64) for water intake from food in men and with (18–25) for water intake from beverages in women) (*p* < 0.0001 where significant).

Consumption was generally lower in the elderly for all types of beverages, except for alcoholic drinks and hot beverages.

Consumption of alcoholic drinks for men and women adults averaged 181.3 mL/day (SE: 1.6) and 72.5 mL/day (SE: 0.4) respectively (5.2% and 2.7% of the energy intakes) and increased to 196.7 mL/day and 84.5 mL/day for elderly men and women respectively (6.3% and 3.5% of the total energy, data not shown).

In Table 5, we compared the water intakes and beverage consumptions between seasons separately for men and women. Total water intake from food and beverages was highest during the summer and lowest in the winter. Difference in TWI between summer and winter was 120 mL for men and 80 mL for women (*p* < 0.0001). Consumption of hot beverages was higher during the winter compared to the three other seasons (*p* < 0.001). Conversely, consumption of milk, caloric soft drinks, diet soft drinks, alcoholic drinks and water were higher during the spring and summer (see Table 5 for significant pairwise comparisons).

**Figure 3:**
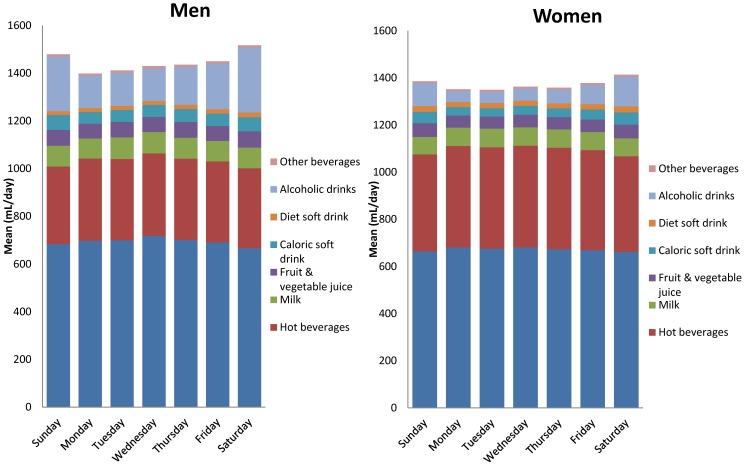
Amount and types of beverages consumed according to day of the week (mean mL/day), separated by gender.

We investigated the influence of the day of the week on the beverage consumption (Figure 3).

The total amount of beverage intake (mL) was higher on Saturdays and on Sundays compared to other days of the week (*p* < 0.0001). There is a higher consumption of alcoholic drinks at weekends (*p* < 0.0001). Both men and women consumed around two times more alcoholic drinks on Saturdays than on a week day. Weekends were also associated with a lower consumption of water and hot beverages, and a higher consumption of caloric soft drink (*p* < 0.0001).

Beverage consumption according to the time of day over a timeline of 24 h was presented in Table 6. The main part of the beverage consumption was concentrated during meal times. Elderly men’s and women’s consumption of beverages was higher than in the other age groups during breakfast (except with group (51–64)) (*p* < 0.0001), but lower during lunch and dinner (*p* < 0.0001).

**Figure 4:**
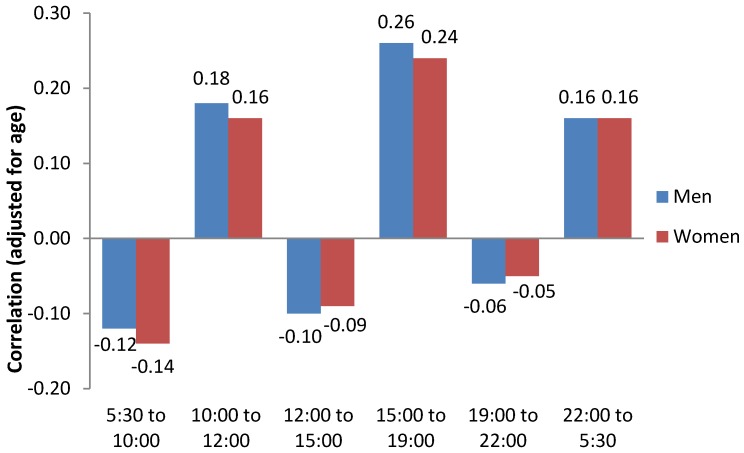
Partial correlations between TWI and beverage consumption in each period, by gender, adjusted for age. Beverage consumption in each period expressed as a percentage of total consumption over 24 h.

Interestingly, we found significant positive correlations between total water intake and a consumption of beverages outside meal times, and significant negative correlations with consumption during meal times (Figure 4).

## 4. Discussion

In this population, TWI was in line with EFSA AI reference values [6]. We found that 61.9% of the TWI came from beverages and 38.1% came from food. The part of the water intake coming from food in our population was then higher than the EFSA estimation for European countries (70%–80% provided by the beverages and 20%–30% coming from food). Differences in percentage of TWI from food were also found in other countries, for example in Ireland (33%) [19] or in China (40%) [20], depending on dietary patterns. Importance of food in TWI should then not be neglected, especially in elderly people, for which we found that the water intake coming from food was higher than in the other age groups.

Similarly to the results found in other previous studies conducted on French adults, water was the first contributor to beverage consumption for both men and women (48.2% for women and 46.6% for men). Water represented 43%–46% of beverage consumption in Bellisle et al. [21], and 49% in Guelinckx et al. [22]. The second contributor was hot beverages (respectively 30.2% and 23.5% for women and men). Those values are comparable to the ones found in Bellisle et al. (20%) and Guelinckx et al. (25%).

The energy impact of caloric soft drinks was small in our population. For the whole sample, caloric soft drinks contributed only 1% of the total kcal. The contribution of caloric soft drinks to energetic intakes was almost equal for men and women. The elderly population had a consumption representing less than 1% of the EI. However, this consumption was higher in the youngest group (18–25 years) in which it represented 2.2% of the total kcal (data not shown). The impact of caloric soft drink consumption on weight gain [23,24,25,26], obesity [27,28,29] and metabolic disorders [30,31,32,33] has been shown in several studies. Large differences between countries have been observed [22] and it was interesting to investigate it in a large population of young French adults. Our results are comparable to those found previously in another smaller French population [21]. In our population, the consumption of caloric soft drinks is globally low. However, for the younger subjects, the consumption of caloric soft drinks was much higher, and this fact may be a problem in the future as excessive consumption of sugary beverages has been linked with the increasing weight of the population [34,35,36].

Our results suggest that in our population, alcoholic drinks had a higher impact on the total energy intake than caloric soft drinks. Alcoholic drinks contributed to 3.3% of the total energy intake (5.2% for men and 2.7% for women). The consumption of alcohol increased with age. However, the consumption of alcohol was mainly concentrated during the week-end days, which may reflect a festive consumption rather than a regular consumption.

Although we found statistically significant differences in TWI according to the seasonality, the difference of 120 g/day for men and 80 g/day for women between water intakes during winter and summer was limited compared to results of previous studies in other European countries [37,38]. However, this result can be explained by the temperate climate in France, with few extreme temperatures. In a Mediterranean country such as Greece [37], a 40% increase in total water intakes was found between winter and summer. In another study with subjects from Germany, Spain and Greece [38], the difference was 200 g/day between winter and summer.

In regard to the day of the week, it was found that the total amount of beverages consumed was higher on Sundays and Saturdays for both men and women, due to a higher consumption of alcoholic drinks during the week end. However, the higher beverages intake during the week-end may not be associated with a better hydration status due to the diuretic effect of alcohol. It should be interesting to have information from hydration biomarkers, and to study their evolution throughout the week.

We found that, in our study population, beverage consumption was concentrated during the meal times. However, in the elderly population, a higher intake was observed during breakfast compared to lunch and dinner. An original result was to find a positive correlation between TWI and consumption of beverages outside meal times. On the contrary, we found a negative correlation with consumption during meal times, especially breakfast. This negative correlation for breakfast could be due to elderly people having a lower daily water intake but a higher water intake during breakfast. This correlation pattern between TWI and time indicates that people with a higher water intake tend to consume beverages regularly throughout the day and not only concentrated during meal times. A major strength of this study is its reliance on the use of a very large population of French adults (Nutrinet-Santé study). The intakes of both food and beverages were estimated precisely using three 24-h records, randomly distributed within a two-week period, including two weekdays and one weekend day. However, our study also suffers from some limitations. For example, even if we assessed the level of physical activity using IPAQ questionnaire at baseline, the actual physical activity level during the day of the 24-h dietary records is unknown. We cannot then evaluate the impact of the physical activity level on TWI. Moreover, caution is also needed when generalizing our results, since the NutriNet-Santé study is a long-term cohort focusing on nutrition and participants are recruited on a voluntary basis, implying that they might have increased health consciousness and interest in nutritional issues as well as a healthier lifestyle.

## 5. Conclusions

The present study gives an overview of the characteristics of water intake in a large population of French adults. TWI was found to be globally in line with public health recommendations. However, we found significant differences in beverage consumption across age groups and seasons. Further research should be directed towards examining the association between hydration status and chronic diseases in different populations to determine the optimal level of water intake. These data will be necessary in order to formulate public health recommendations.

## Figures and Tables

**Table 1:** Statistical description for the whole sample, and by gender.

		Male	Female	Total
Count		**20,636**	**74,303**	**94,939**
Age (year) Means (SE)		47.3 (0.10)	41.7 (0.05)	42.9 (0.04)
Age Group	18–25 (%)	8.7	15.6	14.1
	26–35 (%)	18.0	23.0	21.9
	36–50 (%)	27.2	30.4	29.7
	51–64 (%)	33.3	26.2	27.8
	65–75 (%)	12.8	4.6	6.4
Rate of unemployment (%)		5.0	6.5	6.1
Level of physical activity	Vigorous (%)	36.7	26.3	28.6
	Moderate (%)	32.9	38.3	37.1
	Light (%)	18.9	21.3	20.7
	Unknown (%)	11.5	14.1	13.5
Level of education	Primary (%)	23.1	17.7	18.9
	Secondary (%)	13.7	18.4	17.4
	Post-secondary (%)	63.2	63.9	63.7
Household income	<1000 €/month (%)	8.4	13.0	12.0
	1000–2000 €/month (%)	36.4	39.3	38.7
	≥2000 €/month (%)	48.3	34.6	37.6
	Unknown (%)	6.9	13.1	11.8
Weight (kg) Means (SE)		77.4 (0.09)	63.3 (0.05)	66.4 (0.05)
Height (cm) Means (SE)		176.5 (0.05)	164.1 (0.02)	166.8 (0.03)
BMI (kg/m^2^) Means (SE)		24.8 (0.03)	23.5 (0.02)	23.8 (0.01)
BMI class	Normal weight (%)	58.8	72.6	69.6
	Overweight (%)	32.3	18.3	21.4
	Obese (%)	8.8	9.0	9.0
Waist Circumference * Means (SE)		90.32 (0.16)	79.92 (0.11)	82.83 (0.10)

* Subsample of individuals who had attended the clinical exam, *n* = 16,133.

**Table 2:** Contribution of food and beverages to total water (mL/day) and energy intake (kcal/day) for the whole sample and by gender.

		Contribution to Water Intake (mL/Day)			*p*	Contribution to Energy Intake (kcal/Day)			*p*
		Male	Female	Total		Male	Female	Total	
Count		20,636	74,303	94,939		20,636	74,303	94,939	
All food and drink	Mean (SE)	2251.0 (4.7)	2101.7 (2.4)	2134.2 (2.2)	<0.0001	2250 (3.6)	1783 (1.5)	1884 (1.5)	<0.0001
Food only	%	39.4	37.8	38.1	<0.0001	90.1	92.2	91.7	<0.0001
Beverages only	%	60.6	62.2	61.9	<0.0001	9.9	7.8	8.3	<0.0001
Hot beverages	%	14.6	19.0	18.1	<0.0001	0.3	0.4	0.4	<0.0001
Milk	%	3.6	3.5	3.5	NS	1.9	2.1	2.0	<0.0001
Fruit & vegetable juice	%	2.6	2.4	2.4	<0.0001	1.3	1.3	1.3	0.0003
Caloric soft drink	%	2.3	2.1	2.1	<0.0001	1.0	0.9	1.0	NS
Diet soft drink	%	0.7	1.1	1.0	<0.0001	0.0	0.1	0.1	<0.0001
Alcoholic drinks	%	7.3	3.1	4.0	<0.0001	5.2	2.7	3.3	<0.0001
Water	%	29.2	30.7	30.4	<0.0001	0.0	0.0	0.0	NS
Other beverages	%	0.3	0.3	0.3	NS	0.2	0.2	0.2	0.0003

NS: Not significant; SE: standard error.

**Table 3:** Partial correlations between water intake, energy intake and beverage consumption, adjusted for age, gender, body weight and activity level.

	Total Water (from Food & Beverages)	Water from Beverages	Water from Food	Beverages Weight	Food Weight	Total Energy kcal	Energy from Beverages	Energy from Food
Total water (from food and beverages)		0.91 ***	0.53 ***	0.91 ***	0.54 ***	0.32 ***	0.12 ***	0.30 ***
Water from beverages	0.91 ***		0.19 ***	1.00 ***	0.22 ***	0.24 ***	0.21 ***	0.19 ***
Water from food	0.53 ***	0.19 ***		0.18 ***	0.96 ***	0.32 ***	−0.14 ***	0.38 ***
Beverages weight	0.91 ***	0.999 ***	0.18 ***		0.21 ***	0.25 ***	0.25 ***	0.18 ***
Food weight	0.54 ***	0.22 ***	0.96 ***	0.21 ***		0.51 ***	−0.12 ***	0.58 ***
Total energy kcal	0.32 ***	0.24 ***	0.32 ***	0.25 ***	0.51 ***		0.31 ***	0.95 ***
Energy from beverages	0.12 ***	0.21 ***	−0.14 ***	0.25 ***	−0.12 ***	0.31 ***		0.05 ***
Energy from food	0.30 ***	0.19 ***	0.38 ***	0.18 ***	0.58 ***	0.95 ***	0.05 ***	
(1) Hot beverages (mL)	0.46 ***	0.46 ***	0.19 ***	0.45 ***	0.19 ***	0.06 ***	−0.14 ***	0.10 ***
(2) Milk (mL)	0.03 ***	0.06 ***	−0.02 ***	0.07 ***	−0.02 ***	0.10 ***	0.42 ***	0.003
(3) Fruit & vegetable juice (mL)	0.09 ***	0.11 ***	−0.004	0.12 ***	0.003	0.12 ***	0.32 ***	0.04 ***
(4) Caloric soft drink (mL)	−0.04 ***	0.02 ***	−0.14 ***	0.03 ***	−0.10 ***	0.12 ***	0.27 ***	0.06 ***
(5) Diet soft drink (mL)	0.06 ***	0.06 ***	0.02 ***	0.06 ***	0.01 **	−0.02 ***	−0.01**	−0.02 ***
(6) Alcoholic drinks (mL)	0.08 ***	0.15 ***	−0.10 ***	0.17 ***	−0.09 ***	0.19 ***	0.55 ***	0.04 ***
(7) Water (mL)	0.65 ***	0.70 ***	0.15 ***	0.69 ***	0.17 ***	0.12 ***	−0.04 ***	0.15 ***
(8) Other beverages (mL)	0.06 ***	0.04 ***	0.06 ***	0.04 ***	0.06 ***	0.01 ***	0.06 ***	−0.001
Variety of beverages consumed in day (out of 8)	0.38 ***	0.43 ***	0.05 ***	0.45 ***	0.08 ***	0.25 ***	0.51 ***	0.13 ***

*** Correlation is significant at the <0.0001 level (bilateral); ** Correlation is significant at the 0.01 level (bilateral).

**Table 4:** Results of ANOVA test for total water intake (g/day) and beverage consumption (mL/day), by gender and by age group.

		*Male*								*Female*							
		Age Group						*p*	Pairwise Comparison	Age Group						*p*	Pairwise Comparison
		18–25	26–35	36–50	51–64	65–75	Total			18–25	26–35	36–50	51–64	65–75	Total		
		A	B	C	D	E				A	B	C	D	E			
Base		1793	3709	5614	6873	2647	20,636			11,609	17,130	22,626	19,502	3436	74,303		
Total water intake from food and beverages (mL/day)	Mean (SE)	2108.8 (16.4)	2247.3 (11.8)	2328.2 (9.7)	2269.9 (7.8)	2139.7 (11.3)	2251.0 (4.7)	<0.0001	A = E < B = D < C	1858.7 (5.9)	2073.3 (5.2)	2173.6 (4.5)	2187.8 (4.5)	2102.3 (9.9)	2101.7 (2.4)	<0.0001	A < B = E < C = D
Water from food (mL/day)	Mean (SE)	726.9 (6.4)	779.0 (4.4)	846.7 (3.7)	912.6 (3.4)	923.5 (5.2)	855.9 (1.9)	<0.0001	A < B < C < D = E	640.2 (2.2)	704.2 (1.8)	778.7 (1.6)	855.3 (1.8)	870.7 (4.2)	764.2 (0.9)	<0.0001	A < B < C < D < E
Water from beverages (mL/day)	Mean (SE)	1381.9 (14.1)	1468.3 (10.2)	1481.6 (8.4)	1357.3 (6.5)	1216.2 (9.4)	1395.1 (4.1)	<0.0001	E < D = A < B = C	1218.6 (5.0)	1369.1 (4.5)	1394.9 (3.9)	1332.5 (3.9)	1231.7 (8.3)	1337.5 (2.1)	<0.0001	A = E < D < B < C
Total beverage consumption (mL/day)	Mean (SE)	1439.7 (14.5)	1523.6 (10.4)	1531.8 (8.6)	1406.8 (4.5)	1262.7 (9.6)	1446.0 (4.1)	<0.0001	E < D = A < B = C	1260.1 (5.1)	1406.8 (4.5)	1426.5 (4.0)	1362.4 (3.9)	1261.2 (8.4)	1371.5 (2.1)	<0.0001	A = E < D < B < C
*OF WHICH (mL/day)*																	
Hot beverages	Mean (SE)	152.5 (5.1)	269.8 (4.5)	367.2 (4.2)	387.4 (3.4)	371.0 (5.6)	338.2 (2.1)	<0.0001	A < B < C = E	206.9 (2.5)	356.8 (2.5)	487.8 (2.5)	522.0 (2.6)	498.5 (5.8)	423.2 (1.3)	<0.0001	A < B < C = E < D
									A, B, C < D								
Milk	Mean (SE)	121.2 (3.5)	94.9 (2.3)	84.6 (1.8)	77.3 (1.5)	88.1 (2.6)	87.7 (0.9)	<0.0001	D < C < B < A	101.6 (1.2)	87.8 (1.0)	72.1 (0.8)	61.6 (0.8)	61.5 (1.9)	77.1 (0.4)	<0.0001	D = E < C < B < A
									D < E < A								
Fruit & vegetable juice	Mean (SE)	89.5 (2.7)	83.7 (1.8)	67.1 (1.3)	54.1 (1.0)	42.8 (1.4)	64.6 (0.7)	<0.0001	E < D < C < B = A	74.9 (0.9)	65.9 (0.7)	47.7 (0.5)	41.7 (0.5)	36.4 (1.1)	54.0 (0.3)	<0.0001	E < D < C < B < A
Caloric soft drink	Mean (SE)	132.4 (4.8)	82.5 (2.5)	57.4 (1.8)	29.4 (1.1)	18.2 (1.1)	54.1 (0.9)	<0.0001	E < D < C < B < A	95.0 (1.4)	55.3 (0.8)	28.0 (0.5)	17.2 (0.5)	14.4 (0.9)	41.3 (0.4)	<0.0001	E = D < C < B < A
Diet soft drink	Mean (SE)	22.3 (2.3)	32.3 (1.9)	23.0 (1.4)	7.8 (0.7)	4.5 (0.8)	17.2 (0.6)	<0.0001	E = D < A = C < B	27.1 (0.9)	36.2 (0.9)	22.6 (0.6)	11.2 (0.5)	6.5 (0.8)	22.7 (0.3)	<0.0001	E < D < C < A < B
Alcoholic drinks	Mean (SE)	129.3 (5.0)	172.1 (3.4)	167.1 (2.9)	205.4 (2.8)	196.7 (3.9)	181.3 (1.6)	<0.0001	A < B = C < D = E	57.7 (1.1)	71.8 (0.8)	71.6 (0.8)	80.8 (0.8)	84.5 (1.9)	72.5 (0.4)	<0.0001	A < B = C < D = E
Water	Mean (SE)	782.5 (12.1)	778.7 (8.3)	754.5 (7.0)	637.7 (5.5)	535.0 (7.5)	694.2 (3.4)	<0.0001	E < D < C = B = A	690.2 (4.2)	725.4 (3.6)	688.0 (3.1)	619.1 (3.0)	550.2 (6.4)	672.5 (1.7)	<0.0001	E < D < C = A < B
Other beverages	Mean (SE)	9.8 (1.2)	9.5 (1.0)	10.9 (0.7)	7.2 (0.5)	6.4 (0.8)	8.8 (0.3)	<0.0001	D < C	6.7 (0.4)	7.6 (0.3)	8.8 (0.3)	8.9 (0.3)	9.1 (0.7)	8.2 (0.1)	<0.0001	A < C, D, E, B < D
									E < C								

**Table 5:** Total water intake (g/day) and beverage consumption (mL/day), by season and by gender.

		*Male*						*Female*					
		Season				*p*	Pairwise Comparison	Season				*p*	Pairwise Comparison
		Dec–Jan–Feb	Mar–Apr–May	Jun–Jul–Aug	Sep–Oct–Nov			Dec–Jan–Feb	Mar–Apr–May	Jun–Jul–Aug	Sep–Oct–Nov		
		A	B	C	D			A	B	C	D		
Base		3607	9206	5518	2305			11,969	33,503	19,894	8937		
Total water intake from food and beverages (mL/day)	Mean (SE)	2196.7 (10.7)	2228.3 (7.1)	2317.6 (9.5)	2267.0 (14.1)	<0.0001	A < D	2081.2 (5.9)	2074.9 (3.6)	2154.0 (4.9)	2113.6 (7.0)	<0.0001	A = B < D < C
							A, B, D < C						
Water from food (mL/day)	Mean (SE)	880.2 (4.7)	831.4 (2.8)	865.6 (3.8)	892.7 (6.2)	<0.0001	B < A, C, D	774.8 (2.3)	744.0 (1.4)	782.9 (1.9)	784.3 (2.8)	<0.0001	B < A < C = D
							C < D						
Water from beverages (mL/day)	Mean (SE)	1316.5 (9.0)	1397.0 (6.1)	1452.0 (8.2)	1374.3 (12.0)	<0.0001	A < B = D < C	1306.3 (4.9)	1330.9 (3.1)	1371.0 (4.1)	1329.2 (5.9)	<0.0001	A < B = D < C
Total beverage consumption (mL/day)	Mean (SE)	1365.0 (9.2)	1448.4 (6.2)	1504.3 (8.4)	1423.5 (12.2)	<0.0001	A < B = D < C	1339.4 (5.0)	1365.3 (3.1)	1405.3 (4.2)	1362.5 (5.9)	<0.0001	A < B = D < C
*OF WHICH (mL/day)*													
Hot beverages	Mean (SE)	373.0 (5.1)	332.1 (3.0)	324.5 (3.9)	341.2 (6.2)	<0.0001	B = C = D < A	484.1 (3.6)	409.3 (2.0)	403.7 (2.5)	437.2 (3.8)	<0.0001	B = C < D < A
Milk	Mean (SE)	80.6 (2.2)	89.3 (1.4)	89.4 (1.8)	88.1 (3.0)	<0.0001	B = C = D	70.0 (1.1)	79.3 (0.7)	77.5 (0.9)	77.1 (1.3)	<0.0001	B = C = D
							A < B, A < C						A < B, A < C, A < D
Fruit & vegetable juice	Mean (SE)	64.8 (1.7)	65.6 (1.0)	64.2 (1.3)	61.1 (2.0)	NS		53.1 (0.8)	54.8 (0.4)	53.7 (0.6)	52.9 (0.9)	NS	
Caloric soft drink	Mean (SE)	35.5 (1.7)	58.9 (1.4)	60.5 (1.9)	48.5 (2.4)	<0.0001	A < D < B = C	31.0 (0.8)	45.6 (0.6)	43.2 (0.7)	34.6 (0.9)	<0.0001	A = D < C < B
Diet soft drink	Mean (SE)	11.2 (1.1)	19.0 (1.0)	19.3 (1.3)	14.2 (1.5)	<0.0001	A < B, C	15.5 (0.7)	25.3 (0.5)	23.9 (0.7)	19.9 (0.9)	<0.0001	A < D < B = C
Alcoholic drinks	Mean (SE)	170.5 (3.5)	181.9 (2.4)	191.6 (3.1)	170.9 (4.5)	0.0008	A < C, D < C	71.2 (1.1)	71.1 (0.7)	76.5 (0.8)	70.5 (1.2)	<0.0001	A = B = D < C
Water	Mean (SE)	615.5 (7.3)	694.9 (5.1)	746.1 (6.9)	690.5 (10.0)	<0.0001	A < B = D < C	602.5 (3.8)	672.9 (2.5)	719.4 (3.4)	660.5 (4.7)	<0.0001	A < B = D < C
Other beverages	Mean (SE)	13.9 (1.1)	6.7 (0.4)	8.8 (0.7)	9.1 (1.0)	<0.0001	B < A, C < A, D < A	11.8 (0.5)	7.0 (0.2)	7.4 (0.3)	9.7 (0.5)	<0.0001	B = C < D < A

NS: Non significant.

**Table 6:** Beverage consumption according to time of day (hour interval), by gender and by age group.

	*Male*								*Female*							
	Age Group								Age Group							
Mean amount of beverages (mL/day) consumed between	18–25	26–35	36–50	51–64	65–75	Total	*p*	Pairwise Comparison	18–25	26–35	36–50	51–64	65–75	Total	*p*	Pairwise Comparison
	A	B	C	D	E				A	B	C	D	E			
	1793	3709	5614	6873	2647	20,636			11,609	17,130	22,626	19,502	3436	74,303		
Breakfast 5:30 to 10:00	251.8 (4.5)	320.3 (3.5)	384.4 (2.9)	408.3 (2.6)	405.7 (4.2)	372.1 (1.5)	<0.0001	A < B < C < D = E	246.7 (1.7)	330.3 (1.6)	405.7 (1.5)	434.4 (1.6)	445.1 (3.9)	372.8 (0.8)	<0.0001	A < B < C < D = E
Mid-morning 10:00 to 12:00	104.8 (3.3)	110.6 (2.4)	102.3 (2.1)	69.2 (1.6)	51.0 (2.1)	86.4 (1.0)	<0.0001	E < D < C = A = B	103.1 (1.2)	106.5 (1.1)	98.7 (0.9)	80.4 (1.0)	59.2 (1.9)	94.6 (0.5)	<0.0001	E < D < C < A = B
Lunch 12:00 to 15:00	378.8 (5.2)	394.3 (3.7)	392.4 (3.0)	374.0 (2.4)	346.4 (3.6)	379.5 (1.5)	<0.0001	D < B, D < C	334.4 (1.8)	354.6 (1.5)	341.4 (1.3)	316.7 (1.3)	291.5 (3.0)	334.6 (0.7)	<0.0001	E < D < A < C < B
								E < A, B, C, D								
Snack 15:00 to 19:00	178.3 (4.9)	173.8 (3.4)	174.1 (2.9)	151.5 (2.3)	127.9 (3.1)	161.0 (1.4)	<0.0001	E < D < A = B = C	162.0 (1.6)	184.8 (1.5)	189.6 (1.3)	181.5 (1.3)	160.2 (2.9)	180.7 (0.7)	<0.0001	A = E < B = C = D
Dinner 19:00 to 22:00	419.5 (6.0)	429.2 (4.3)	403.6 (3.2)	349.8 (2.6)	289.2 (3.6)	377.0 (1.6)	<0.0001	E < D < C = A	350.9 (2.0)	366.9 (1.7)	337.6 (1.4)	294.7 (1.4)	253.4 (3.0)	331.3 (0.8)	<0.0001	E < D < C < A < B
								C, D, E < B								
Night 22:00 to 5:30	107.3 (4.6)	95.2 (2.8)	75.2 (1.9)	53.6 (1.4)	42.4 (1.9)	70.2 (1.0)	<0.0001	E < D < C < B < A	63.3 (1.2)	64.0 (1.0)	53.5 (0.8)	54.9 (0.8)	52.0 (1.8)	57.8 (0.4)	<0.0001	C < A, C < B, D < A
																D < B, E < A, E < B
